# Decipher correlation patterns post prostatectomy: initial experience from 2 342 prospective patients

**DOI:** 10.1038/pcan.2016.38

**Published:** 2016-08-30

**Authors:** R B Den, M Santiago-Jimenez, J Alter, M Schliekelman, J R Wagner, J F Renzulli II, D I Lee, C G Brito, K Monahan, B Gburek, N Kella, G Vallabhan, F Abdollah, E J Trabulsi, C D Lallas, L G Gomella, T L Woodlief, Z Haddad, L L C Lam, S Deheshi, Q Wang, V Choeurng, M du Plessis, J Jordan, B Parks, H Shin, C Buerki, K Yousefi, E Davicioni, V R Patel, N L Shah

**Affiliations:** 1 Department of Radiation Oncology, Sidney Kimmel Medical College of Thomas Jefferson University, Philadelphia, PA, USA; 2 GenomeDx Biosciences Inc., Vancouver, BC, Canada; 3 Urologic Oncology and Minimally Invasive Surgery, Hartford Healthcare Medical Group, Hartford, CT, USA; 4 Section of Minimally Invasive Urology, The Warren Alpert Medical School of Brown University, Providence, RI, USA; 5 Division of Urology, Penn Presbyterian Medical Center, Philadelphia, PA, USA; 6 Arizona Urology Specialists, Scottsdale, AZ, USA; 7 Penn Urology, Philadelphia, PA, USA; 8 The Urology and Prostate Institute, San Antonio, TX, USA; 9 Lubbock Urology, Lubbock, TX, USA; 10 Vattikuti Urology Institute, Henry Ford Hospital, Detroit, MI, USA; 11 Department of Urology, Sidney Kimmel Medical College of Thomas Jefferson University, Philadelphia, PA, USA; 12 Global Robotics Institute, Celebration, FL, USA; 13 Piedmont Health Care, Atlanta, GA, USA

## Abstract

**Background:**

Currently, there are multiple commercially available RNA-based biomarkers that are Medicare approved and suggested for use by the National Comprehensive Cancer Network guidelines. There is uncertainty as to which patients benefit from genomic testing and for whom these tests should be ordered. Here, we examined the correlation patterns of Decipher assay to understand the relationship between the Decipher and patient tumor characteristics.

**Methods:**

De-identified Decipher test results (including Decipher risk scores and clinicopathologic data) from 2 342 consecutive radical prostatectomy (RP) patients tested between January and September 2015 were analyzed. For clinical testing, tumor specimen from the highest Gleason grade was sampled using a 1.5 mm tissue punch. Decipher scores were calculated based on a previously locked model. Correlations between Decipher score and clinicopathologic variables were computed using Spearman's rank correlation. Mixed-effect linear models were used to study the association of practice type and Decipher score. The significance level was 0.05 for all tests.

**Results:**

Decipher score had a positive correlation with pathologic Gleason score (PGS; *r*=0.37, 95% confidence interval (CI) 0.34−0.41), pathologic T-stage (*r*=0.31, 95% CI 0.28−0.35), CAPRA-S (*r*=0.32, 95% CI 0.28−0.37) and patient age (*r*=0.09, 95% CI 0.05-0.13). Decipher reclassified 52%, 76% and 40% of patients in CAPRA-S low-, intermediate- and high-risk groups, respectively. We detected a 28% incidence of high-risk disease through the Decipher score in pT2 patients and 7% low risk in pT3b/pT4, PGS 8−10 patients. There was no significant difference in the Decipher score between patients from community centers and those from academic centers (*P*=0.82).

**Conclusions:**

Although Decipher correlated with baseline tumor characteristics for over 2 000 patients, there was significant reclassification of tumor aggressiveness as compared to clinical parameters alone. Utilization of the Decipher genomic classifier can have major implications in assessment of postoperative risk that may impact physician-patient decision making and ultimately patient management.

## Introduction

The management of men with prostate cancer is continuously evolving. Significant controversy exists with regard to the appropriate utilization and timing of adjuvant vs salvage therapies. Novel RNA-based genomic signatures are aiding patients and physicians in selection of primary therapy,^1, 2^ as well as with postoperative treatment options.^3, 4, 5, 6, 7, 8, 9, 10, 11^ While these assays provide important prognostic information, only the Decipher prostate cancer classifier has been shown to be predictive^12^ and consideration for its use in the post-radical prostatectomy (RP) setting has been suggested by the National Comprehensive Cancer Network guidelines.^13^ Decipher has also been validated for prediction of metastasis among patients who experience biochemical recurrence and in the salvage radiotherapy setting post RP.^10, 14^ The Decipher post-op test is marketed to patients with adverse clinical or pathologic findings (e.g., pathologic stage T2 with positive margins, any pT3 disease or biochemical recurrence). Currently, there is uncertainty as to which patients benefit from genomic testing and thus for whom these tests should be ordered. Hence, we examined the commercial correlation patterns of the Decipher assay to understand the relationship between the genomic classifier score and patients' tumor characteristics and to gain insight into the potential impact on altering post-operative therapy decisions for this patient population.

## Materials and methods

### Study cohort

We identified 2 504 consecutive RP patients from academic and community health centers for whom the Decipher test was ordered between 1 January and 1 September 2015. The tests were ordered by physicians at the originating institutions for patient management in the post-prostatectomy setting. Only patient samples that passed microarray quality control (QC) metrics were included in this study (*n*=2 342). Microarray QC was verified using Affymetrix power tools^15^ and was performed by running the specimens through an internal QC pipeline that flags specimens that fail microarray QC metrics as described previously.^4, 5^ The proportion of patient samples that failed microarray QC metrics was 5.9% (162 samples), of which 64.8% had insufficient signal (low percent present of probes on the microarray) and the rest did not meet the other required QC metrics for hybridization and overall signal quality. The microarray QC failed samples were distributed across 45/303 ordering centers (1−5 failed samples per center) and the average monthly QC failure rate was 5.3%. None of the patients in the current study were part of previously reported validation studies of Decipher. All patient-related data were de-identified and study researchers were blinded to patient identities and did not have access to personally identifiable health information.

### Specimen selection and processing

Dedicated genitourinary pathologists from originating institutions graded all tumors using the International Society of Urological Pathology 2005 Gleason grading criteria.^16^ For lab processing, only the formalin-fixed paraffin-embedded block submitted for Decipher testing was reviewed. The submitted formalin-fixed paraffin-embedded block for molecular analysis contained the index prostate cancer lesion with the highest stage and tumor grade, and was sampled using a 1.5 mm tissue punch tool provided in the specimen kit.

### Calculation of CAPRA-S and Decipher

CAPRA-S score was derived from a Cox regression equation using six variables: preoperative PSA, pathologic Gleason score (PGS), surgical margin status, extra-prostatic extension, seminal vesicle invasion and lymph node invasion.^17^

The expression values for the 22 pre-specified biomarkers that constitute Decipher were extracted from the normalized data matrix and entered into the locked random forest algorithm with tuning and weighting parameters defined as reported previously.^5^ The Decipher read-out is a continuous risk score between 0 and 1, with higher scores indicating a greater probability of metastasis and prostate cancer-specific mortality.^7^ Validated low- (<0.45), intermediate- (0.45−0.60) and high-risk (>0.60) groups of Decipher were used for categorical analyses.^5^

### Statistical analyses

Descriptive statistics of variables focused on medians and interquartile ranges (IQR) or frequencies and proportions as appropriate. To compare clinico-pathologic variables across practice types, Fisher's exact test and analysis of variance *F* test were used for categorical and continuous variables, respectively. Correlations between Decipher score and age categories, PGS, pathologic stage and CAPRA-S were computed using Spearman's rank correlation. Linear regression analysis was used to compare Decipher scores in patients with and without tertiary Gleason pattern. A mixed-effect linear model was fitted to study the association between practice type and Decipher score. In this analysis, institution was modelled as a random effect to adjust for its possible bias on Decipher variations. All statistical tests were two-sided and had a significance level of 0.05. Analyses were performed in R v3.1 (R Foundation, Vienna, Austria).

## Results

### Patient characteristics and utilization patterns in the ordering centers

Demographic, clinical and pathological characteristics of the study cohort are provided in Table 1. Breakdown of study cohort by practice type is provided in Supplementary Table 1. Overall, 10.6% of patients came from academic practice centers compared to 89.3% from community centers. Median patient age at RP was 66 years (IQR, 60−69). Median time between RP and ordering of Decipher was 3.6 months (IQR, 0.8−14.8). Fifty-four percent of patients had positive surgical margins and 60.6% had pT3 or higher disease; 7.9%, 38.4%, 28.8%, 10.7% and 14.0% had PGS 3+3, 3+4, 4+3, 8 and 9−10, respectively. Only 14.9% (*n*=349) of tests were performed for patients with pT2R0 disease and, of these, 21 (0.9%) had Gleason 6 disease. Decipher tests ordered by practitioners in academic centers were for higher-risk patients as judged by the greater proportion of Gleason 9−10 tumors (18.5% vs 13.5%, *P*<0.03) and patients with lymph node invasion (6.8% vs 2.1%, *P*<0.001), whereas in the community-based practice a higher proportion of tests were ordered for older men (median 66 vs 64 years old, *P*<0.001) who had higher rate of positive surgical margins (55.2% vs 46.6%, *P*<0.01) (Supplementary Table 1).

Overall, of the 303 ordering centers, 172 (56.8%), 67 (22.1%), 51 (16.8%) and 13 (4.3%) ordered 1−3, 4-9, 10−39 and 40 or more Decipher tests, respectively (Supplementary Table 2). These groups of ordering centers represent 11.5%, 16.1%, 37.2% and 35.1% of the entire study cohort, respectively.

### Correlation of Decipher score with PGS, pathologic stage and CAPRA-S

**Figure 1:**
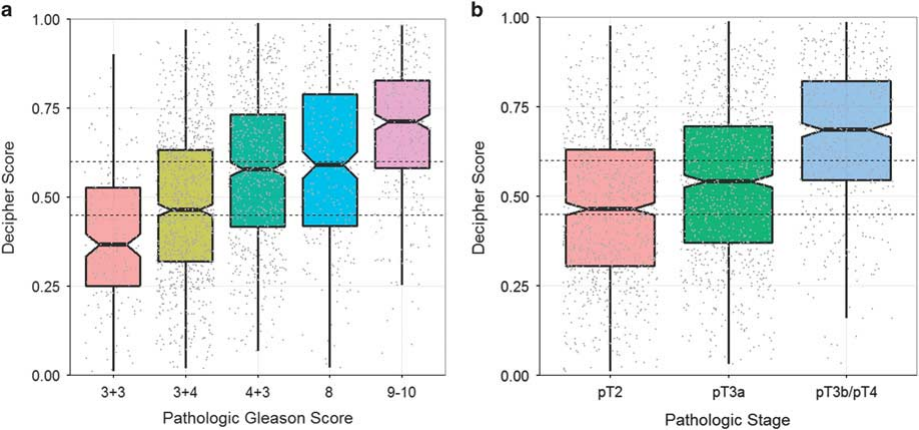
Correlation of Decipher score with (**a**) pathologic Gleason score; (**b**) pathologic stage.

**Figure 2:**
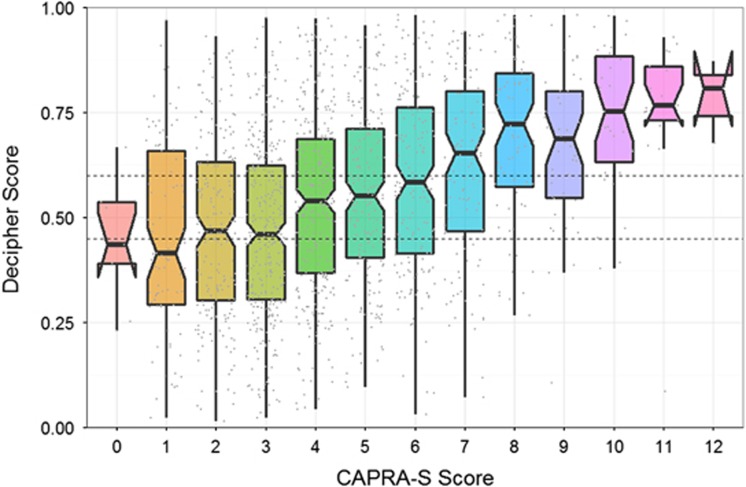
Correlation of Decipher score and CAPRA-S.

Figure 1 provides the correlation of Decipher score with PGS and pathologic stage. Decipher score had a positive Spearman's correlation of 0.37 (95% confidence interval (CI) 0.34-0.41) with PGS (Figure 1a). Similar results were observed between Decipher score and pathologic stage as patients with favorable pathology had significantly lower Decipher scores (Figure 1b; Spearman's correlation of 0.31; 95% CI 0.28−0.35). We also assessed the association between Decipher score and CAPRA-S. This analysis showed that Decipher had a positive Spearman's correlation of 0.32 (95% CI: 0.28−0.37) with CAPRA-S (Figure 2).

### Decipher reclassifies patients assigned to risk groups by PGS, pathologic stage and CAPRA-S

Decipher classification by PGS and pathologic stage is provided in Table 2. Of the 2243 (95.8%) patients with available pathologic data, Decipher classified 35.8%, 23.1% and 41.1% as low-, intermediate- and high-risk, respectively. Decipher provided independent information and reclassified patients within PGS and pathologic stage categories (Table 2). For instance, 10% of patients with PGS 3+3 and pT2 stage were classified as high-risk, while 7% of patients with PGS 8−10 and pT3b/pT4 disease were classified as low-risk by Decipher.

**Figure 3:**
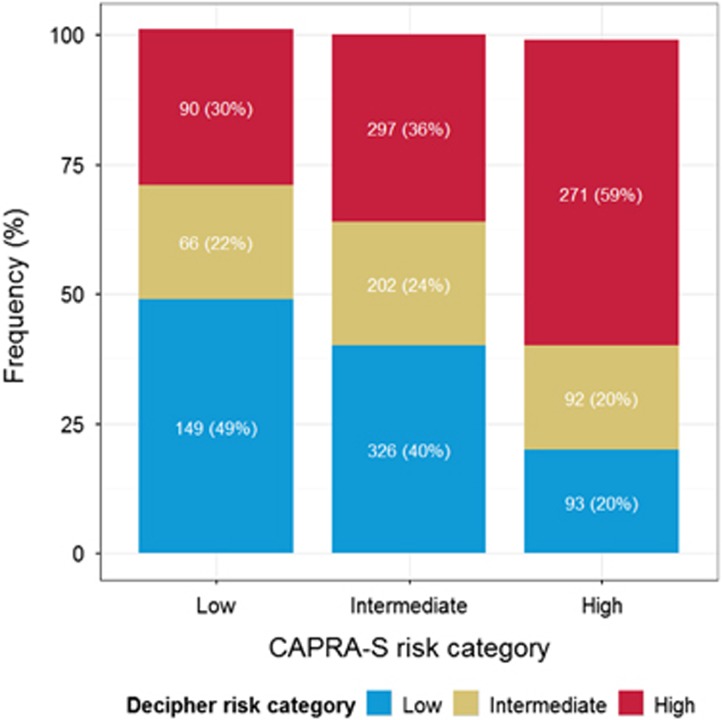
Decipher stratification within low, intermediate, and high CAPRA-S risk groups.

Risk stratification of patients by CAPRA-S to Decipher is shown in Supplementary Figure 1. Of the 2 342 patients in the study, 1 586 (67.7%) had available CAPRA-S score. Of these, 19.2%, 52.0% and 28.8% were classified as low-, intermediate- and high-risk, respectively. In contrast, Decipher classified 35.8%, 22.7% and 41.5% of these patients as low-, intermediate- and high-risk, respectively. Decipher classification within CAPRA-S score categories is provided in Figure 3. Decipher reclassified 52%, 76% and 40% of patients in CAPRA-S low-, intermediate- and high-risk groups, respectively.

### Association of Decipher score with tertiary 5 Gleason pattern

**Figure 4:**
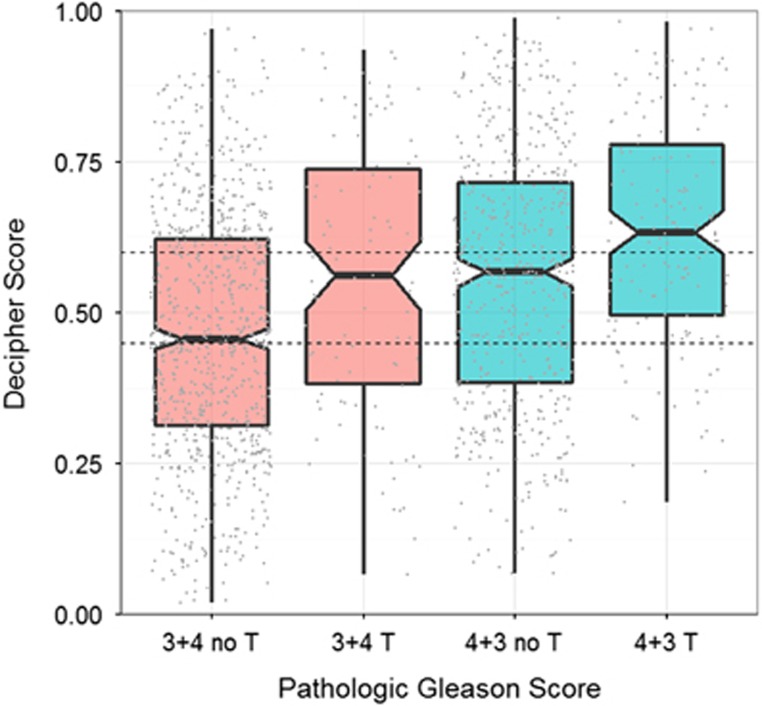
Association of Decipher score with tertiary Gleason pattern 5 among patients with pathologic Gleason score 3+4 and 4+3. T refers to tertiary Gleason pattern 5.

Among patients with PGS 3+4 and 4+3, 99 (11.0%) and 155 (23.0%) had a tertiary Gleason pattern 5, respectively. Among patients with PGS 3+4 and 4+3, those with a tertiary pattern had +0.09 (95% CI: 0.05−0.14) and +0.08 (95% CI: 0.05−0.12) higher Decipher score on average, respectively (Figure 4). The linear model with PGS and tertiary pattern as predictors and Decipher score as a response variable showed that patients with tertiary Gleason pattern 5 had +0.09 (95% CI 0.06−0.12; *P*<0.001) higher Decipher scores on average than patients without tertiary pattern after adjusting for PGS (Supplementary Table 3).

### Decipher score in community vs academic practice centers

The multivariable linear mixed effect model with institution as a random effect showed no significant difference (*P*=0.82) in Decipher scores between academic and community practice centers after adjusting for clinico-pathologic variables (Supplementary Table 4).

### Association of Decipher score with age categories

**Figure 5:**
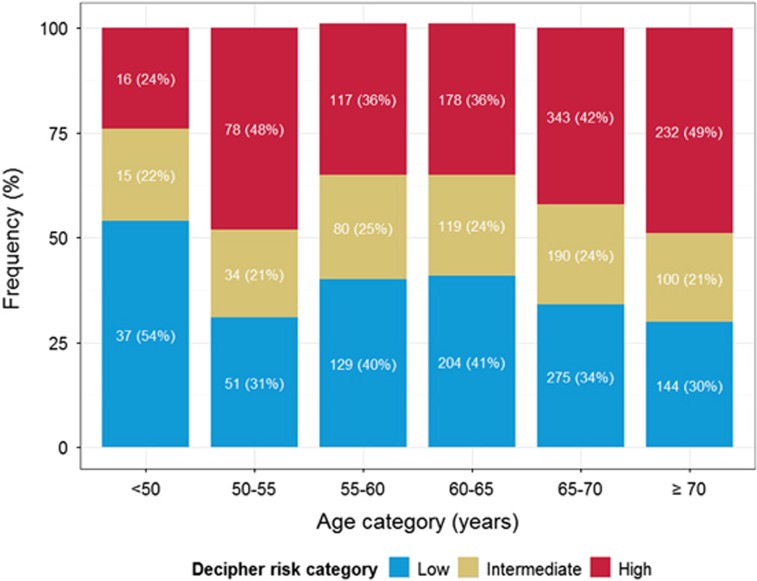
Decipher stratification within age categories.

Decipher score had a positive Spearman's correlation of 0.09 (95% CI 0.05-0.13, *P*<0.001) with patient age (Figure 5). In this analysis, 54% of patients aged <50 were categorized as low risk while only 30% of patients who were 70 or older were categorized as low Decipher risk. However, after adjusting for PSA, PGS, surgical margin status, extra-prostatic extension, seminal vesicle invasion and lymph node invasion, the association between age at prostatectomy and Decipher score was not statistically significant.

## Discussion

The delivery and timing of post-operative radiation therapy is controversial within the medical community.^18^ While multiple randomized studies^19, 20, 21^ have demonstrated an improvement in biochemical recurrence with immediate (adjuvant) post-prostatectomy radiation for men with either pathologic T3 disease or positive margins, an improvement in overall survival has been shown in only one study.^22^ Further, 50% of patients who were randomized to observation following prostatectomy never developed further disease progression. This has led many to advocate for offering post-operative radiation therapy only in the setting of a detectable PSA (salvage) with close monitoring of PSA, most often at a PSA >0.2 ngml^−1^.^23^

We have recently demonstrated that integration of a genomic classifier can distinguish patients who would benefit from adjuvant radiation therapy from those who can be safely treated with salvage radiation therapy.^12^ There was no statistically significant difference in cumulative incidence of metastasis for men classified as low risk by Decipher regardless of whether they had received adjuvant vs salvage radiation therapy. However, there was a clinically meaningful and statistically significant decrease in cumulative incidence of metastasis with administration of adjuvant as opposed to salvage radiation therapy for men classified as Decipher high risk.

Multiple studies have established that, when provided hypothetical cases, physicians will alter management decisions based on genomic data.^24, 25, 26^ Further, decision curve analysis demonstrates that, when compared to scenarios where no prediction model would be used for a post-operative radiation treatment decision (that is, ‘treat all' or ‘treat none'), genomic classifier-based models had a higher net benefit than clinical models across a wide range of decision threshold probabilities.^27^ These studies emphasize the importance of genomic markers in making patient management decisions. Given the potential for toxicity with post-operative therapy as radiation, hormonal or chemotherapy balanced against the potential for cure, more information will assist in patient decision-making. Forthcoming, prospective studies are necessary and vital to ensure that these findings are valid as bias can be reduced.

Our current study demonstrates several salient features. First, the majority of patients were referred from community practices, demonstrating the adoption of the Decipher genomic classifier in current practice within the US.^28^ Second, we believe this is the first demonstration in prospective usage that the genomic classifier correlates with both pathologic stage and PGS and it provides unique patient-specific information. Third, the high percentages of positive surgical margins and pT3 or higher disease in this study are reflective of the marketed indications of the Decipher test (that is, the presence of adverse pathologic or clinical findings). Fourth, only a small fraction of tests (0.9%) were ordered in node negative patients with pT2R0 disease and pathologic Gleason 6. For patients in whom post-operative radiation is not currently indicated (pathologic T2 and margin negative), 25% of men had high-risk disease as determined by the genomic classifier. Conversely, in patients typically considered for adjuvant radiation therapy (pathologic T3 or greater and Gleason score 8−10), 18% of men were classified as having low-risk disease. Finally, assuming that all men with pT3 or positive margins received adjuvant radiation therapy, utilization of the genomic classifier would reduce usage in the low-risk population by approximately 36%.

Our study is not without limitations. First, we do not have long-term follow-up on the patients analyzed to determine the actuarial metastatic incidence. Furthermore, while robust, the genomic classifier provides an estimate for incidence of metastatic disease for a given patient over a 5- and 10-year period, which is not absolute. Third, the predicted incidence of metastasis may decrease with more aggressive therapy being offered earlier to patients at higher risk of disease;^29^ however, no post-operative treatment information is currently available for these patients. Fourth, while the assay informs clinical decision making, selection of further therapy, by either the physician or the patient, was not captured in this initial experience and will be reported in subsequent analyses.

## Conclusions

While Decipher correlated with baseline tumor characteristics for over 2 000 patients whose tumor specimens underwent genomic testing, there was significant reclassification of tumor aggressiveness as compared to risk assessment based on clinical parameters alone. Patients and providers can determine their willingness to accept a given threshold risk of metastasis and determine whether genomic testing would be beneficial. Given the wide distribution of risk classification and potential of the genomic classifier score to reclassify patients and thereby alter management between observation, adjuvant or salvage radiation therapy following prostatectomy, genomic testing could be a helpful tool within the post-operative setting.

## Figures and Tables

**Table 1:** Clinical and pathological characteristics of the study cohort

*Variables*	*Study cohort*
No. of patients	2342
*Practice type*	
Academic	249 (10.6%)
Community	2092 (89.3%)
Unknown	1 (0%)

*Age at RP*	
Median (range)	66 (40–84)
IQR (Q1, Q3)	(60–69)

*Time between RP and ordering of Decipher (months)*	
Median (range)	3.6 (0.1–102)
IQR (Q1, Q3)	(0.8–14.8)

*Pre-op PSA (ng*^*−1*^*ml)*	
Median (range)	6.4 (0–150)
IQR (Q1, Q3)	(4.7–9.6)
<10 ng ml^−1^	1265 (54.0%)
10-20 ng ml^−1^	277 (11.8%)
>20 ng ml^−1^	110 (4.7%)
Unknown	690 (29.5%)

*Extra-prostatic extension,* n *(%)*	
Present	1297 (55.4%)

*Seminal vesicle invasion*,^a^ n *(%)*	
Present	504 (21.5%)

*Surgical margin*,^a^ n *(%)*	
Positive	1271 (54.3%)

*Lymph node invasion*,^a^ n *(%)*	
Positive	61 (2.6%)

*Pathologic Gleason score,* n *(%)*	
6	186 (7.9%)
7	
(3+4)	900 (38.4%)
(4+3)	674 (28.8%)
8	251 (10.7%)
9–10	328 (14.0%)
Unknown	3 (0.1%)

*Pathological stage,* n *(%)*	
T2R0	349 (14.9%)
T2R1	564 (24.1%)
T3a	903 (38.6%)
T3b	501 (21.4%)
T4	14 (0.6%)
Unknown	11 (0.5%)

Abbreviations: IQR, interquartile range; RP, radical prostatectomy.

a Unknown seminal vesicle invasion, surgical margin and lymph node invasion status for 11, 6 and 95 patients, respectively.

**Table 2:** Decipher classification with pathologic Gleason score and pathologic stage risk groups

	*Gleason score*^a^														
	*3+3*			*3+4*			*4+3*			*8-10*			*Total*		
*Decipher*	*Low*	*Int*	*High*	*Low*	*Int*	*High*	*Low*	*Int*	*High*	*Low*	*Int*	*High*	*Low*	*Int*	*High*
pT2	74 (64%)	30 (26%)	12 (10%)	221 (56%)	85 (22%)	87 (22%)	71 (39%)	44 (24%)	68 (37%)	46 (30%)	37 (24%)	71 (46%)	412 (49%)	196 (23%)	238 (28%)
pT3a	29 (64%)	9 (20%)	7 (16%)	150 (45%)	78 (23%)	104 (31%)	91 (30%)	89 (29%)	126 (41%)	39 (22%)	38 (21%)	103 (57%)	309 (36%)	214 (25%)	340 (39%)
pT3b/pT4	6 (50%)	3 (25%)	3 (25%)	29 (24%)	30 (25%)	60 (50%)	21 (14%)	30 (20%)	97 (66%)	14 (7%)	32 (16%)	148 (76%)	70 (15%)	95 (20%)	308 (65%)
Total	109 (63%)	42 (24%)	22 (13%)	400 (47%)	193 (23%)	251 (30%)	183 (29%)	163 (26%)	291 (46%)	99 (19%)	107 (20%)	322 (61%)	791 (36%)	505 (23%)	886 (41%)

Ninety nine patients with unknown pathologic stage or pathologic Gleason score or lymph node data were excluded.

a Excluding lymph node-positive patients.
