# Supplementary material for: Decipher correlation patterns post prostatectomy: initial experience from 2 342 prospective patients
Source: Prostate Cancer Prostatic Dis. 2016 Aug 30;19(4):374–9. doi: 10.1038/pcan.2016.38 (PMC5133268; doi:10.1038/pcan.2016.38)
Supplement: Supplementary Information [file pcan201638x1.doc]

**Supplemental material**

**FIGURE LEGENDS**

**Figure S1**. Stratification of patients in the study by Decipher and CAPRA-S risk groups

**TABLE LEGENDS**

**Table S1.** Clinical and pathological characteristics of the study cohort by practice setting

**Table S2.** Utilization patterns in ordering centers

**Table S3.** Linear regression models of Decipher score in PGS 3+4 and 4+3 with and without tertiary Gleason pattern 5

**Table S4.** Multivariable linear mixed effect model to compare Decipher score in community vs. academic practice center

**Figure S1**. Stratification of patients in the study by Decipher and CAPRA-S risk groups

**
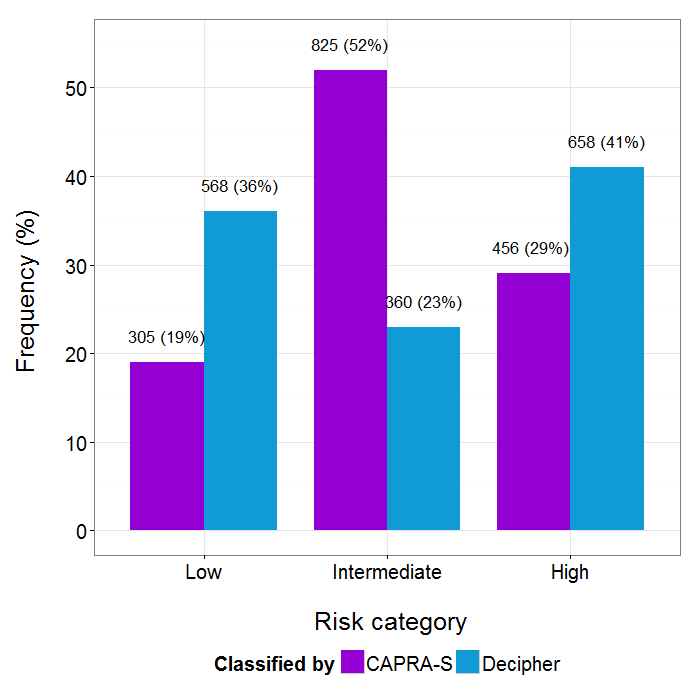
**

**Table S1.** Clinical and pathological characteristics of the study cohort by practice setting

| **Variables** | **Academic** | **Community** | **p** |
| --- | --- | --- | --- |
| No. patients* | 249 | 2 092 |  |
| **Age at RP** |  |  | <0.001 |
| Median (Range) | 64 (42-78) | 66 (40-84) |  |
| IQR (Q1,Q3) | (58 - 68) | (61 - 69) |  |
| **Time between RP and ordering of Decipher (months)** |  |  | 0.67 |
| Median (Range) | 3.3 (0.1 - 102) | 3.7 (0.1-101.2) |  |
| IQR (Q1,Q3) | (1.1 - 13.4) | (0.7 – 15.0) |  |
| **Pre-op PSA (ng/mL)** |  |  | 0.45 |
| Median (Range) | 6.4 (0 - 116) | 6.4 (0 - 150) |  |
| IQR (Q1,Q3) | (4.6 - 10.2) | (4.8 - 9.6) |  |
| <10 ng/mL | 133 (53.4%) | 1131 (54.1%) |  |
| 10-20 ng/mL | 35 (14.1%) | 242 (11.6%) |  |
| >20 ng/mL | 12 (4.8%) | 98 (4.7%) |  |
| Unknown | 69 (27.7%) | 621 (29.7%) |  |
| **Extra-prostatic Extension, n (%)** |  |  | 0.06 |
| Present | 152 (61%) | 1 145 (54.7%) |  |
| **Seminal Vesicle Invasion*, n (%)** |  |  | 0.06 |
| Present | 65 (26.1%) | 438 (20.9%) |  |
| **Surgical margin*, n (%)** |  |  | 0.01 |
| Positive | 116 (46.6%) | 1 155 (55.2%) |  |
| **Lymph node invasion*, n (%)** |  |  | <0.001 |
| Positive | 17 (6.8%) | 44 (2.1%) |  |
| **Pathologic Gleason score, n (%)** |  |  | 0.03 |
| 6 | 13 (5.2%) | 173 (8.3%) |  |
| 7 |  |  |  |
| (3+4) | 81 (32.5%) | 818 (39.1%) |  |
| (4+3) | 81 (32.5%) | 593 (28.3%) |  |
| 8 | 28 (11.2%) | 223 (10.7%) |  |
| 9-10 | 46 (18.5%) | 282 (13.5%) |  |
| Unknown | 0 (0%) | 3 (0.1%) |  |
| **Pathological stage, n (%)** |  |  | 0.03 |
| T2R0 | 41 (16.5%) | 308 (14.7%) |  |
| T2R1 | 43 (17.3%) | 521 (24.9%) |  |
| T3a | 97 (39%) | 806 (38.5%) |  |
| T3b | 63 (25.3%) | 437 (20.9%) |  |
| T4 | 3 (1.2%) | 11 (0.5%) |  |
| Unknown | 2 (0.8%) | 9 (0.4%) |  |
| * Unknown practice type, seminal vesicle invasion, surgical margin and Lymph node invasion status for 1, 11, 6 and 95 patients, respectively  Abbreviations: IQR= interquartile range PSA= prostate specific antigen, RP= radical prostatectomy | | | |

**Table S2. Utilization patterns in ordering centers**

| **Variables** | **Study Cohort** |
| --- | --- |
| No. ordering centers | 303 |
| No. ordering physicians | 407 |
| **No. ordering physicians by center** |  |
| 1 | 238 (78.5%) |
| 2 | 36 (11.9%) |
| 3 | 17 (5.6%) |
| 4-6 | 12 (2.9%) |
| **No. Decipher tests ordered by center** |  |
| Median (Range) | 3 (1 - 137) |
| IQR (Q1,Q3) | (1 - 8) |
| 1-3 | 172 (56.8%) |
| 4-9 | 67 (22.1%) |
| 10-39 | 51 (16.8%) |
| 40 or more | 13 (4.3%) |
| **No. Decipher tests ordered by physician** |  |
| Median (Range) | 2 (1 - 137) |
| IQR (Q1,Q3) | (1 - 5) |
| 1-2 | 223 (54.8%) |
| 3-5 | 87 (21.4%) |
| 6-19 | 71 (17.4%) |
| 20 or more | 26 (6.4%) |
|  | |

**Table S3.** Linear regression models of Decipher score in PGS 3+4 and 4+3 with and without tertiary Gleason pattern 5

| **Variable** | **Average increase in Decipher Score (95% CI)** | **p** |
| --- | --- | --- |
| Path Gleason Score 4+3 (vs. 3+4) | 0.086 (0.065, 0.107) | <0.001 |
| Tertiary 5 Gleason pattern (vs. none) | 0.088 (0.06, 0.116) | <0.001 |

**Table S4. Multivariable linear mixed effect model to compare Decipher score in community vs. academic practice center**

| **Multivariable model** | | |
| --- | --- | --- |
| **Variable** | **Average increase in Decipher score* (95% CI)** | **p*** |
| Pre-op PSA (per 0.01 ng/mL) | 0.024 (-0.074, 0.121) | 0.64 |
| Pathologic Gleason 3+4 (vs. 3+3) | 0.087 (0.045, 0.128) | <0.001 |
| Pathologic Gleason 4+3 (vs. 3+3) | 0.163 (0.121, 0.206) | <0.001 |
| Pathologic Gleason 8 (vs. 3+3) | 0.182 (0.133, 0.231) | <0.001 |
| Pathologic Gleason 9-10 (vs. 3+3) | 0.257 (0.21, 0.304) | <0.001 |
| Surgical margins | 0.001 (-0.019, 0.021) | 0.88 |
| Extra-prostatic extension | 0.044 (0.024, 0.065) | <0.001 |
| Seminal vesicle invasion | 0.099 (0.073, 0.124) | <0.001 |
| Lymph node invasion | 0.033 (-0.025, 0.091) | 0.27 |
| Community vs. Academic | -0.004 (-0.039, 0.031) | 0.82 |
